# Fault diagnosis of anti-friction bearings based on Bi-dimensional ensemble local mean decomposition and optimized dynamic least square support vector machine

**DOI:** 10.1038/s41598-023-44996-6

**Published:** 2023-10-18

**Authors:** Zhengqiang Xiong, Chang Han, Guorong Zhang

**Affiliations:** 1 School of Mechanical and Electrical Engineering, Wuhan Business University, Wuhan, 430056 China; 2 School of Electronic Information, Wuhan University, Wuhan, 430072 China

**Keywords:** Engineering, Mathematics and computing

## Abstract

In order to ensure the normal operation of rotating equipment, it is very important to quickly and efficiently diagnose the faults of anti-friction bearings. Hereto, fault diagnosis of anti-friction bearings based on Bi-dimensional ensemble local mean decomposition and optimized dynamic least square support vector machine (LSSVM) is presented in this paper. Bi-dimensional ensemble local mean decomposition, an extension of ensemble local mean decomposition from one-dimensional signal processing to Bi-dimensional signal processing, is used to extract the features of anti-friction bearings. Moreover, an optimized dynamic LSSVM is used to fault diagnosis of anti-friction bearings. The experimental results show that Bi-dimensional ensemble local mean decomposition is superior to Bi-dimensional local mean decomposition, optimized dynamic LSSVM is superior to traditional LSSVM, and the proposed Bi-dimensional ensemble local mean decomposition and optimized dynamic LSSVM method is effective for fault diagnosis of anti-friction bearings.

## Introduction

Anti-friction bearings are one of the most important components of mechanical equipments. The failure of anti-friction bearings may cause abnormal operation of mechanical equipments, and even cause serious economic losses and casualties^1–4^. Therefore, in order to ensure the normal operation of rotating equipment, it is very important to quickly and efficiently diagnose the faults of anti-friction bearings^5,6^. Machine learning-based fault diagnosis model is relatively widespread, such as, the deep variational autoencoder^7,8^, multiscale deep belief network^9^, hybrid deep learning^10^ and the stacked denoising autoencoder, and so on.

The features of the vibration signals of anti-friction bearings are the key to obtaining the excellent diagnosis results. The traditional feature extraction methods include wavelet transform, empirical mode decomposition, local mean decomposition, and so on^11,12^. Local mean decomposition is a new adaptive time–frequency analysis method which can adaptively decompose nonlinear and non-stationary complex signals into several physically significant PF components and a residual component^13^. Recently, local mean decomposition has been widely in the fault diagnosis of rotating machinery, for instance, Sharma et al. applied local mean decomposition to extract the features of rotary machine^14^. Ensemble local mean decomposition involves adding different Gaussian white noise to the preprocessed bearing signal multiple times before performing local mean decomposition. The PF component decomposed by ensemble local mean decomposition can overcome the aliasing of signal time–frequency distribution caused by signal intermittency. The Bi-dimensional ensemble local mean decomposition is an extension of ensemble local mean decomposition from one-dimensional signal processing to Bi-dimensional signal processing, and time–frequency images of the bearing signal can be obtained by Bi-dimensional ensemble local mean decomposition.

Moreover, an optimized dynamic LSSVM is used to fault diagnosis of anti-friction bearings. LSSVM can simplify the training process by transforming the quadratic programming problem in support vector machine to a linear problem^15,16^. Recently, LSSVM has been widely in the fault diagnosis of rotating machinery, for instance, Islam et al. applied least-square support vector machines to fault diagnosis of rolling-element bearings^17^. Dynamic LSSVM can increase the dynamic increase and decrease process of training samples, effectively utilize the symmetric positive definite property of kernel expansion matrix, and simplify the solution of Lagrange multipliers in the dynamic learning process. In dynamic LSSVM, the penalty parameter and kernel parameter are determined by Cauchy Particle swarm optimization whose inertia weight is expressed by the standard Cauchy density function. The experimental results show that Bi-dimensional ensemble local mean decomposition is superior to Bi-dimensional local mean decomposition, and optimized dynamic LSSVM is superior to traditional LSSVM.

## Bi-dimensional ensemble local mean decomposition

Local mean decomposition (LMD) is a new adaptive time–frequency analysis method which can adaptively decompose nonlinear and non-stationary complex signals into several physically significant PF components and a residual component. Find all local maximum and minimum points $s_{i}$ of the original signal $x(t)$, calculate the mean of adjacent two extreme points, use the sliding average method for smoothing, obtain the local mean function, and calculate the envelope estimation value $e_{i}$ through the local mean points:

1 $$ e_{i} = {{\left| {s_{i} - s_{i + 1} } \right|} \mathord{\left/ {\vphantom {{\left| {s_{i} - s_{i + 1} } \right|} 2}} \right. \kern-0pt} 2} $$

The first PF component of $x(t)$ is the product of the envelope signal $e_{1} (t)$ and the pure frequency modulation signal $r_{1n} (t)$, which is expressed as follows:

2 $$ PF_{1} (t) = e_{1} (t)r_{1n} (t) $$

$x(t)$ is decomposed into *n* PF components and a residual component $v(t)$, which is expressed as follows:

3 $$ x(t) = \sum\limits_{j = 1}^{n} {PF_{j} } (t) + v(t) $$

Ensemble local mean decomposition (ELMD) is an improvement on traditional local mean decomposition. The essence of ELMD is to perform local mean decomposition on the signal after adding different Gaussian white noise, and calculate the mean of several PF components obtained each time as the final result. The specific calculation process is as follows:

1. Add different Gaussian white noise to the denoised signal $x(t)$:
  4 $$ y(t) = x(t) + \varepsilon (t) $$
  where $\varepsilon (t)$ is the Gaussian white noise.
2. Perform LMD decomposition on $y(t)$ to obtain *n* PF components and a residual component $v(t)$, which are expressed as follows:
  5 $$ y(t) = \sum\limits_{j = 1}^{n} {PF_{j} } (t) + v(t) $$
3. Calculate the mean of the PF component and residual obtained as follows:
  6 $$ \left\{ {\begin{array}{*{20}l} {\tilde{P}_{j} (t) = \sum\limits_{j = 1}^{N} {{{PF_{jn} (t)} \mathord{\left/ {\vphantom {{PF_{jn} (t)} N}} \right. \kern-0pt} N}} } \hfill \\ {\tilde{v}(t) = \sum\limits_{j = 1}^{N} {{{v_{j} (t)} \mathord{\left/ {\vphantom {{v_{j} (t)} N}} \right. \kern-0pt} N}} } \hfill \\ \end{array} } \right. $$
  where $\tilde{P}_{j} (t)$ and $\tilde{v}(t)$ are the results of ELMD.

Bi-dimensional ensemble local mean decomposition is an extension of ensemble local mean decomposition from one-dimensional signal processing to Bi-dimensional signal processing. The essence of the Bi-dimensional ensemble local mean decomposition algorithm is to extract ensemble local extreme points of Bi-dimensional image signals to filter and obtain multiple Bi-dimensional production functions with certain physical significance and a trend image. Bi-dimensional ensemble local mean decomposition algorithm is based on the image data itself, which can decompose the Bi-dimensional image into a finite number of Bi-dimensional production function components and a residual component based on the image data to characterize the different frequency features of the image.

The original image can be represented as the sum of the *BPF* component and the remaining signal, which can be expressed as follows:

7 $$ y(k,l) = \sum\limits_{j = 1}^{n} {BPF_{j} } (k,l) + v(k,l) $$

**Figure 1:**
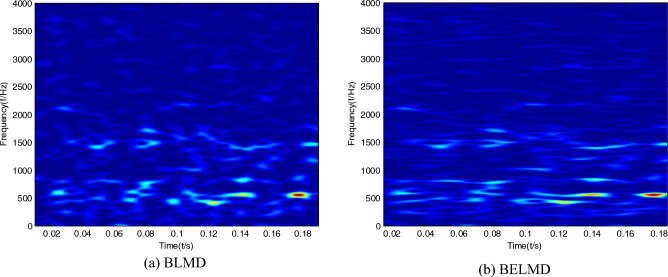
Time–frequency images of the sample with normal condition.

**Figure 2:**
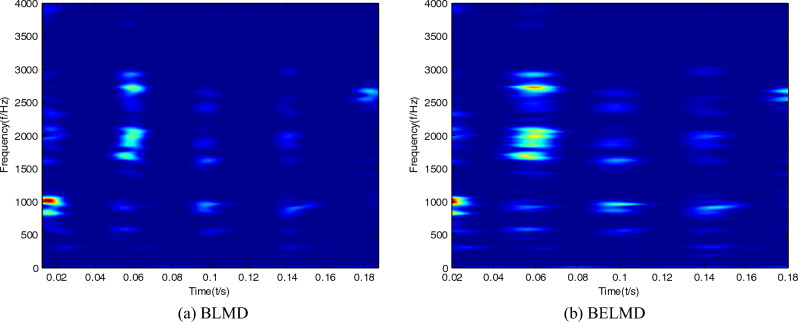
Time–frequency images of the sample with inner race fault.

**Figure 3:**
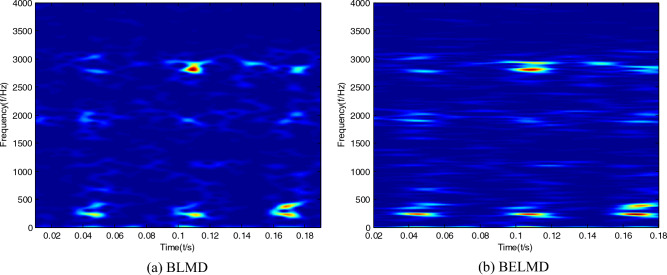
Time–frequency images of the sample with outer race fault.

Time–frequency images of the bearing signal can be obtained by Bi-dimensional ensemble local mean decomposition. In order to embody the superiority of Bi-dimensional ensemble local mean decomposition compared with Bi-dimensional ensemble local mean decomposition, time–frequency images can be respectively obtained by Bi-dimensional local mean decomposition, and Bi-dimensional ensemble local mean decomposition can be shown in Figs. 1, 2 and 3. It can be seen that the features of time–frequency images obtained by Bi-dimensional ensemble local mean decomposition are clearer than those obtained by Bi-dimensional local mean decomposition.

## Optimized dynamic LSSVM

In LSSVM, the Lagrangian function is constructed as follows^18^:

8 $$ L\left( {w,b,\varepsilon_{i} ,\alpha_{i} } \right) = \frac{1}{2}\left( {\left\| w \right\|^{2} + C\sum\limits_{i = 1}^{n} {\varepsilon_{i}^{2} } } \right) - \sum\limits_{i = 1}^{n} {\alpha_{i} \left[ {y_{i} \left( { < w^{T} \cdot \phi (x_{i} ) > + b} \right) - 1 + \varepsilon_{i} } \right]} $$

where $\varepsilon_{i}$ is the error ,$C$ is the penalty parameter, and $\alpha_{i}$ is the Lagrange multiplier.

Partially differentiate the Lagrangian function, and eliminate $\varepsilon_{i}$, $w$, then,

9 $$ y_{i} \left[ {\sum\limits_{j = 1}^{n} {\alpha_{j} y_{j} K(x_{i} ,x_{j} ) + b} } \right] = 1 - \frac{{\alpha_{i} }}{C} $$

where $K(x_{i} ,x_{j} )$ is the kernel function.

In dynamic LSSVM, Eq. (9) can be changed into the follows:

10 $$ y_{i} \left[ {\sum\limits_{j = 1}^{n} {\sum\limits_{k = 1}^{m} {\alpha^{\prime}_{j} y_{j} } \beta_{k} K(x_{i,k} ,x_{j,k} ) + b} } \right] = 1 - \frac{1}{C}\sum\limits_{k = 1}^{m} {\alpha^{\prime}_{i,k} } $$

Then, the dynamic LSSVM is obtained as follows:

11 $$ g(x) = sign\left( {\sum\limits_{i = 1}^{n} {\sum\limits_{k = 1}^{m} {y_{i} \alpha^{\prime}_{i,k} } K(x_{i,k} ,x_{k} ) + b} } \right) $$

In dynamic LSSVM, the penalty parameter and kernel parameter are determined by Cauchy Particle swarm optimization. Particle swarm optimization is a type of stochastic global optimization algorithm derived from the social behavior of birds flocking^19,20^. Update the position and velocity of each particle as follows:

12 $$ \left\{ {\begin{array}{*{20}l} {v_{i,j} (t + 1) = \omega \cdot v_{i,j} (t) + c_{1} \cdot rand \cdot (pbest_{i,j} - p_{i,j} (t)) + c_{2} \cdot rand \cdot (gbest - p_{i,j} (t))} \hfill \\ {p_{i,j} (t + 1) = p_{i,j} (t) + v_{i,j} (t + 1)} \hfill \\ \end{array} } \right. $$

where $\omega$ is the inertia weight,$pbest_{i,j}$ is the best previous experience of individual particle, $gbest$ is the global best experience,$c_{1}$ and $c_{2}$ are the constants, and $rand$ is the random number from 0 to 1.

The Cauchy distribution can be used as a mutation operator, and the standard Cauchy density function is expressed as follows:

13 $$ R(k) = \frac{1}{{\pi (k^{2} + 1)}} $$

In Cauchy Particle swarm optimization, the inertia weight is expressed by the standard Cauchy density function, then, the position and velocity of each particle to search for the optimal solution is expressed as follows:

14 $$ \left\{ {\begin{array}{*{20}l} {v_{i,j} (t + 1) = R(k) \cdot v_{i,j} (t) + c_{1} \cdot rand \cdot (pbest_{i,j} - p_{i,j} (t)) + c_{2} \cdot rand \cdot (gbest - p_{i,j} (t))} \hfill \\ {p_{i,j} (t + 1) = p_{i,j} (t) + v_{i,j} (t + 1)} \hfill \\ \end{array} } \right. $$

**Figure 4:**
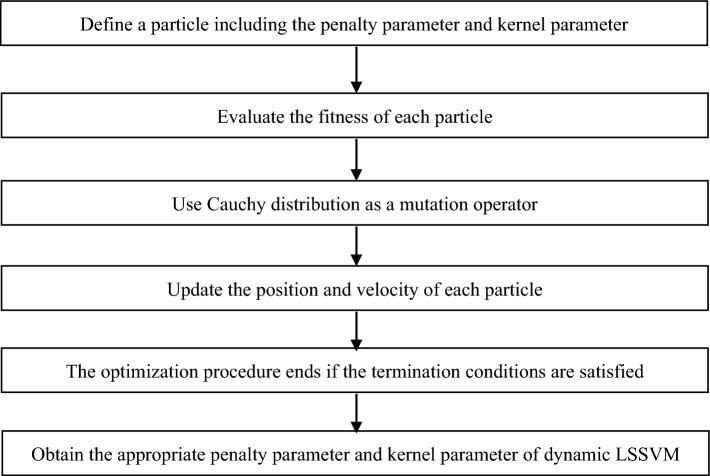
The flowchart of determining the penalty parameter and kernel parameter of dynamic LSSVM.

Cauchy particle swarm optimization is used to determine the penalty parameter and kernel parameter of dynamic LSSVM. As shown in Fig. 4, define a particle including the penalty parameter and kernel parameter, evaluate the fitness of each particle, update the position and velocity of each particle according to Eq. (14), then, the optimization procedure ends if the termination conditions are satisfied.

## Experimental testing and results

**Figure 5:**
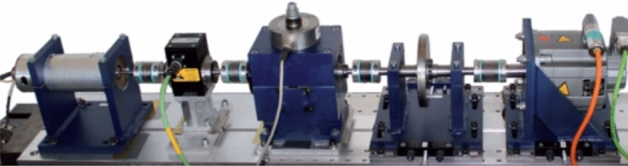
The test rig of “Bearing Fault Dataset” of Paderborn.

**Figure 6:**
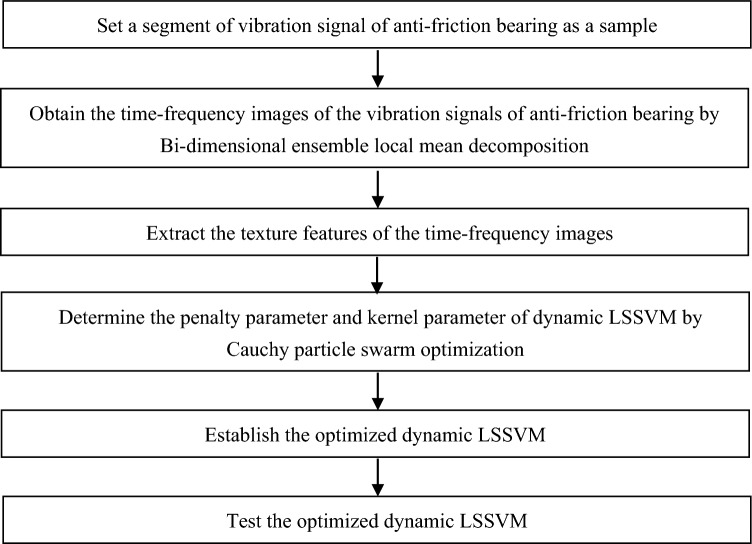
The flowchart of fault diagnosis of anti-friction bearings based on Bi-dimensional ensemble local mean decomposition and optimized dynamic least square support vector machine.

The experimental data derives from “Bearing Fault Dataset” of Paderborn. The test rig of “Bearing Fault Dataset” of Paderborn is shown in Fig. 5. The test rig is equipped with a NICE bearing, and the parameters of the bearing and experimental condition are shown in the literature^21^. The flowchart of fault diagnosis of anti-friction bearings based on Bi-dimensional ensemble local mean decomposition and optimized dynamic least square support vector machine is given in Fig. 6. In the process of determining the penalty parameter and kernel parameter of dynamic LSSVM by Cauchy particle swarm optimization, set the ranges of the penalty parameter and kernel parameter of dynamic LSSVM to [0.01 10000], [0.1 100], respectively, $c_{1}$ is set to 2, and $c_{2}$ is also set to 2.

The main faults of anti-friction bearing are inner race fault, and outer race fault. We use 300 samples with multiple different loads as the experimental samples, among which 180 samples containing normal condition, inner race fault, and outer race fault with multiple different loads are used as the training samples and others are used as the testing samples. In order to testify the superiority of Bi-dimensional ensemble local mean decomposition compared with Bi-dimensional local mean decomposition, and the superiority of optimized dynamic LSSVM compared with traditional LSSVM, the hybrid method of Bi-dimensional local mean decomposition and optimized dynamic LSSVM (BLMD-ODLSSVM), the hybrid method of Bi-dimensional local mean decomposition and traditional LSSVM (BLMD-LSSVM), and traditional LSSVM are respectively used to compared with the hybrid method of Bi-dimensional ensemble local mean decomposition and optimized dynamic LSSVM (BELMD-ODLSSVM).

**Figure 7:**
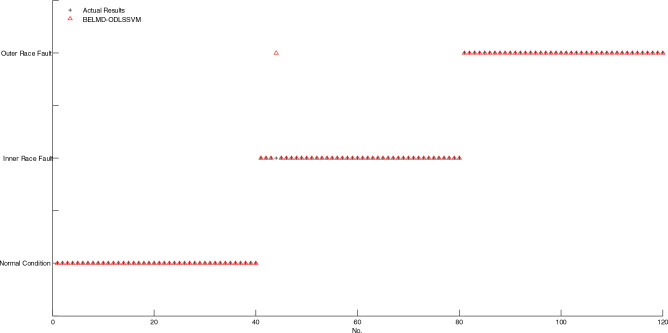
The classification results of BELMD-ODLSSVM.

**Figure 8:**
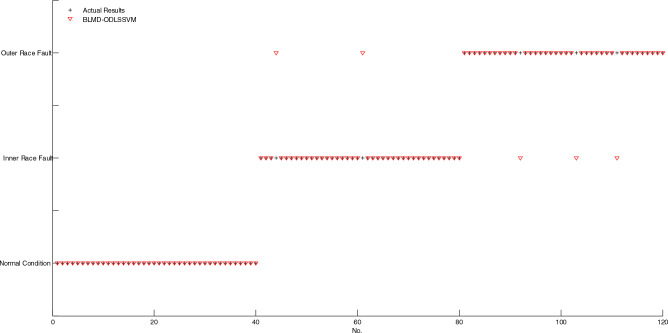
The classification results of BLMD-ODLSSVM.

**Figure 9:**
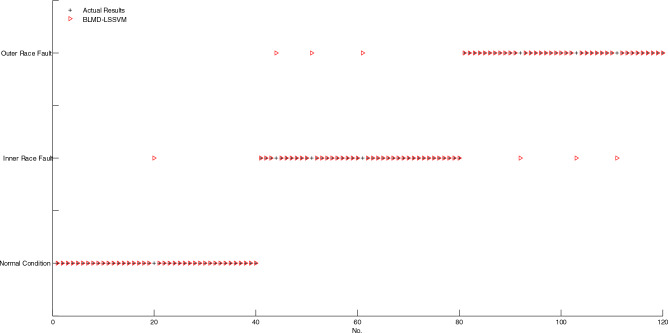
The classification results of BLMD-LSSVM.

**Figure 10:**
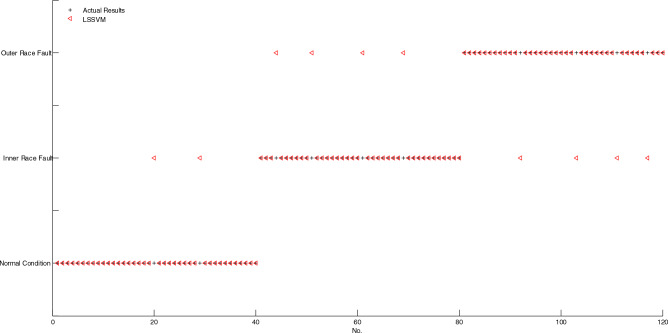
The classification results of LSSVM.

**Figure 11:**
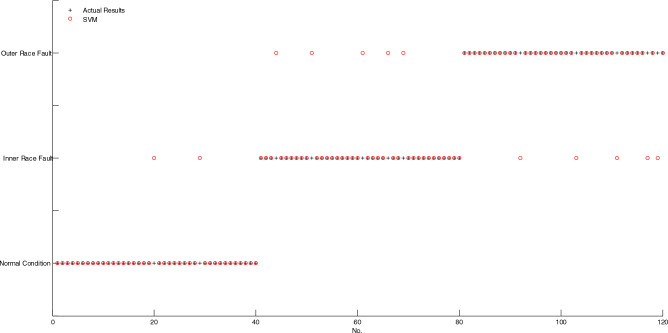
The classification results of SVM.

As shown in Fig. 7, if two markings of actual result and diagnosis result don't coincide, then, the corresponding sample is misclassified, conversely, the corresponding sample is correctly classified. It is obvious that only one sample is misclassified by using BELMD-ODLSSVM. As shown in Fig. 8, the markings of diagnosis results of five samples don't coincide with the markings of actual results, which indicates that five samples are misclassified by using BLMD-ODLSSVM. As shown in Fig. 9, the markings of diagnosis results of seven samples don't coincide with the markings of actual results, which indicates that seven samples are misclassified by using BLMD-LSSVM. As shown in Fig. 10, the markings of diagnosis results of ten samples don't coincide with the markings of actual results, which indicates that ten samples are misclassified by using traditional LSSVM. As shown in Fig. 11, the markings of diagnosis results of twelve samples don't coincide with the markings of actual results, which indicates that twelve samples are misclassified by using SVM.

As shown in Table 1, the classification accuracy of BELMD-ODLSSVM is 99.17%, the classification accuracy of BLMD-ODLSSVM is 95.83%, the classification accuracy of BLMD-LSSVM is 94.17%, the classification accuracy of traditional LSSVM is 91.67%, and the classification accuracy of SVM is 90%, which demonstrates that BELMD-ODLSSVM is the most classification methods among the five methods. Moreover, the classification results between BELMD-ODLSSVM and BLMD-ODLSSVM indicate that Bi-dimensional ensemble local mean decomposition is superior to Bi-dimensional local mean decomposition. The classification results between BLMD-ODLSSVM and BLMD-LSSVM indicate that optimized dynamic LSSVM is superior to traditional LSSVM.

**Table 1:** The classification accuracy of anti-friction bearing among the five methods.

Method	The number of misclassification	The number of correct classification	Accuracy (%)
BELMD-ODLSSVM	1	119	99.17
BLMD-ODLSSVM	5	115	95.83
BLMD-LSSVM	7	113	94.17
LSSVM	10	110	91.67
SVM	12	108	90

## Conclusions

The contributions and conclusions of this paper are described as follows:

1. Ensemble local mean decomposition involves adding different Gaussian white noise to the preprocessed bearing signal multiple times before performing local mean decomposition. The Bi-dimensional ensemble local mean decomposition is an extension of ensemble local mean decomposition from one-dimensional signal processing to Bi-dimensional signal processing, and time–frequency images of the bearing signal can be obtained by Bi-dimensional ensemble local mean decomposition. The classification accuracy of BELMD-ODLSSVM is 99.17%, the classification accuracy of BLMD-ODLSSVM is 95.83%, which demonstrates that Bi-dimensional ensemble local mean decomposition is superior to Bi-dimensional local mean decomposition.
2. An optimized dynamic LSSVM is used to fault diagnosis of anti-friction bearings. Dynamic LSSVM can increase the dynamic increase and decrease process of training samples, effectively utilize the symmetric positive definite property of kernel expansion matrix, and simplify the solution of Lagrange multipliers in the dynamic learning process. In dynamic LSSVM, the penalty parameter and kernel parameter are determined by Cauchy Particle swarm optimization whose inertia weight is expressed by the standard Cauchy density function. The classification accuracy of BLMD-ODLSSVM is 95.83%, the classification accuracy of BLMD-LSSVM is 94.17%, the classification accuracy of traditional LSSVM is 91.67%, which demonstrates that optimized dynamic LSSVM is superior to traditional LSSVM in the fault diagnosis of anti-friction bearings.

## Data Availability

The data that support the findings of this research are available from the corresponding author upon reasonable request.
